# Development and Psychometric Evaluation of the Transtheoretical Model-Based Sustainable Nutrition Behavior Scale (TTM-SNBS) for Adolescents

**DOI:** 10.3390/nu17223516

**Published:** 2025-11-11

**Authors:** Ozlem Muslu, Pervin Demir, Zeynep Caferoglu Akin, Oznur Basdas

**Affiliations:** 1 Department of Nutrition and Dietetics, Institute of Health Sciences, Erciyes University, Kayseri 38280, Türkiye; ozlemmuslu@yiu.edu.tr; 2 Department of Nutrition and Dietetics, Faculty of Health Sciences, Yuksek Ihtisas University, Ankara 06530, Türkiye; 3 Department of Biostatistics and Medical Informatics, Faculty of Medicine, Ankara Yildirim Beyazit University, Ankara 06010, Türkiye; pervin.demr@gmail.com; 4 Department of Nutrition and Dietetics, Faculty of Health Sciences, Erciyes University, Kayseri 38280, Türkiye; 5 Department of Health Sciences, University of York, York YO10 5DD, UK; 6 Department of Nursing, Faculty of Health Sciences, Erciyes University, Kayseri 38280, Türkiye; obasdas@erciyes.edu.tr

**Keywords:** sustainable nutrition, public health, transtheoretical model, adolescents, behavior change, scale development, validity

## Abstract

**Background/Objectives**: Sustainable nutrition is essential for human and planetary health. The Transtheoretical Model-based Sustainable Nutrition Behavior Scale (TTM-SNBS) was developed to assess sustainable nutrition behaviors and the dynamic processes of behavior change in adolescents. **Methods**: The item pool was generated through literature review and expert consultation using the Delphi technique (10 experts, three rounds). Construct validity was assessed using exploratory and confirmatory factor analyses. Reliability was evaluated through Cronbach’s alpha and test–retest methods. External validity was examined through correlations with the Sustainable and Healthy Eating Behaviors Scale (SHEBS), following COSMIN standards. The study included 512 adolescents aged 14–18 years (54% female) from public high schools in Ankara, Türkiye. **Results**: Initially, 75 items were developed through expert evaluation. After preliminary testing, two items with low and negative correlations were removed; 73 were retained for validation. Factor analyses and refinement produced a 45-item final validated scale comprising one stage of change item and five subscales: cognitive processes (14 items), behavioral processes (18 items), decisional balance—pros (4 items), decisional balance—cons (4 items), and self-efficacy (4 items). Items showed good discrimination (>0.27). Cronbach’s alpha values ranged from 0.67 to 0.93, and fit indices were acceptable (χ^2^/*df* = 2.8–4.2; RMSEA = 0.045–0.065). External validity was supported by significant positive correlations with the SHEBS. **Conclusions**: The TTM-SNBS is the first psychometrically validated, theory-based instrument to assess sustainable nutrition behaviors in adolescents. It offers a reliable and valid instrument to support future research and interventions promoting sustainable dietary practices.

## 1. Introduction

Global population growth, environmental change, and the needs of future generations have made sustainable development a global priority. The 17 Sustainable Development Goals (SDGs) adopted by the United Nations emphasize the central role of nutrition in health, the environment, the economy, and society [1]. According to the World Health Organization (WHO) and the Food and Agriculture Organization (FAO), sustainable and healthy nutrition refers to dietary patterns that support optimal growth and health throughout life, have a low environmental impact, and are accessible, affordable, safe, equitable, and culturally acceptable [2]. Factors such as poverty, food insecurity, food safety, equality, and educational quality influence a population’s nutritional status [1].

Adolescents, defined by WHO as individuals aged 10–19 years, represent 1.2 billion people worldwide [3]. This life stage is characterized by rapid physical, cognitive, and social development. It represents a critical period for addressing nutritional needs and establishing sustainable dietary behaviors that contribute to lifelong health benefits [4]. As outlined in the WHO’s *Global Acceleration Action for the Health of Adolescents (AA-HA!)* report [3], adolescent health initiatives support the SDGs in three key ways: promoting healthy living today, enabling healthier living in adulthood, and improving the well-being of future generations [3,4].

In recent years, several instruments have been developed to assess sustainable dietary behaviors [5,6,7,8,9,10,11,12]. These tools primarily focus on (i) comprehensive assessment of healthy and sustainable eating behaviors [9,10,13], (ii) measuring knowledge, attitudes, and behaviors related to sustainable eating [11], and (iii) evaluating current knowledge levels and general attitudes [5,7,12]. However, most existing instruments capture only current attitudes and behaviors, overlooking the dynamic, stage-based nature of behavior change—an aspect widely recognized as central to health behavior research. This limitation highlights a gap in the literature and underscores the need for theory-driven measures that incorporate behavioral change frameworks.

**Figure 1:**
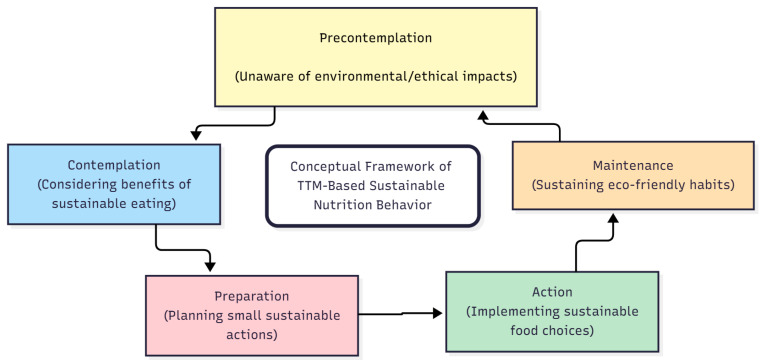
Conceptual framework of TTM-based sustainable nutrition behavior. The diagram illustrates the correspondence between sustainable nutrition behaviors—integrating environmental (e.g., food waste reduction, plant-based choices) and ethical dimensions (e.g., conscious purchasing)—and the stages of the Transtheoretical Model (TTM).

The Transtheoretical Model (TTM), developed by Dr. James Prochaska and colleagues, conceptualizes the change in five stages (precontemplation, contemplation, preparation, action, and maintenance) and includes the components of the change process, decision balance, and self-efficacy [14,15]. Individuals progress through distinct psychosocial processes that facilitate behavior change at different stages [15,16,17]. The conceptual alignment between TTM stages and processes and sustainable eating behaviors, integrating environmental and ethical dimensions, is illustrated in Figure 1.

The present study aimed to develop and validate the Transtheoretical Model-based Sustainable Nutrition Behavior Scale (TTM-SNBS) for adolescents. To our knowledge, this is the first psychometrically validated instrument grounded in TTM principles. The TTM-SNBS makes a novel contribution to the systematic evaluation of sustainable nutrition behaviors among adolescents.

## 2. Materials and Methods

This methodological study was conducted to develop and validate the TTM-SNBS. The data were collected at a single time point, and descriptive statistics were calculated for demographic characteristics of individuals.

### 2.1. Study Design

The development and validation of the TTM-SNBS instrument were conducted in three stages: the development of scale items, the development of the scale itself, and the evaluation of the scale [18]. The reporting of the study was conducted in accordance with the updated COSMIN (COnsensus-based Standards for the selection of health Measurement INstruments) guidelines [19].

### 2.2. Ethical Considerations

The study was approved by the Erciyes University Social and Human Sciences Ethics Committee (approval date: 29 November 2022, number: 549) and conducted in accordance with the principles outlined in the Declaration of Helsinki. It was part of a doctoral dissertation titled Investigation of the Effect of Transtheoretical Model-Based Nutrition Education on Sustainable Nutrition Behavior in Adolescents.

### 2.3. Item Development

The item pool was created through a comprehensive literature review in October and November 2022. A systematic search strategy was applied using international databases (Google Scholar, PubMed, Scopus, and PsycINFO) and national databases such as the National Thesis Center of the Council of Higher Education (Türkiye). The search included the keywords ‘transtheoretical model’, ‘behavior change’, ‘healthy eating’, and ‘stage of change’.

The review included both English and Turkish sources without any language restrictions. Accordingly, existing behavior change scales [20,21,22,23,24,25,26,27,28] and studies focusing on behaviors related to sustainable nutrition and health [2,10,13,29,30,31,32,33,34] were examined. The items were evaluated in terms of conceptual structure, wording, and content, and were subsequently adapted to the concept of sustainable nutrition. To ensure cultural relevance and comprehensibility, Turkish-adapted TTM scales [20,23,26,35] and national theses based on behavior change scales [36,37,38,39,40] were also reviewed.

This process initially resulted in a scale draft consisting of 98 items, including 39 cognitive, 39 behavioral, 14 decisional balance, and six self-efficacy items, as well as 1 stage of change question. These reflected the basic structures of the TTM. The draft version was first reviewed by four faculty members (two from nutrition and dietetics, including one with public health expertise, and two from nursing with model-based research experience).

Subsequently, expert evaluation using the Delphi technique was conducted to ensure the content validity of the draft scale. Details of the Delphi procedure are presented in Section 2.4.1 (Content Validity), including details such as the number of experts, inclusion criteria, and consensus threshold.

### 2.4. Scale Development Process

#### 2.4.1. Content Validity

The TTM-SNBS draft form, comprising an item pool of 99 items, was evaluated by 10 field experts using the Delphi technique. The expert panel consisted of academics with 8–25 years of professional experience, including two full professors specializing in sustainable nutrition and in scale development/adaptation. All experts had prior research or publication experience related to behavioral change, sustainability, or psychometric evaluation. The experts provided a rating for each item, categorizing it as follows: ‘Item is appropriate’, ‘Item is appropriate but can be changed’, or ‘Item is not appropriate’. The experts who indicated the option ‘The item is appropriate but requires alteration’ were invited to propose an enhancement. The content validity of each item was assessed by comparing the expert scores with the Content Validity Ratio (CVR) values suggested by Ayre and Scally (2014) and adjusted according to the number of experts [41]. Responses were collected and managed at Ankara Yildirim Beyazit University Statistical Consultancy Application and Research Centre using REDCap (Research Electronic Data Capture) electronic data capture tools. REDCap is a secure, web-based software platform [42,43].

The Delphi process consisted of three rounds. After each round, the CVR for each item was recalculated, with a value greater than 0.80 considered statistical evidence of consensus [41]. Items that reached consensus in the first round were excluded from subsequent rounds, while revised items were re-evaluated in the second and third rounds.

The scale items on which the experts reached consensus were subsequently reviewed by a linguist for comprehensibility, grammar, and punctuation. To assess the clarity of the items, the revised form was administered to a sample of 10 students aged between 13 and 15. Students were instructed to read and respond to each item individually. No negative feedback was received regarding the items [44]. After the pretest, the scale was finalized.

#### 2.4.2. Preliminary Administration of the TTM-SNBS

The finalized scale was administered to 50 randomly selected adolescents who met the inclusion criteria. The duration of administration and the consistency of responses were analyzed.

**Figure 2:**
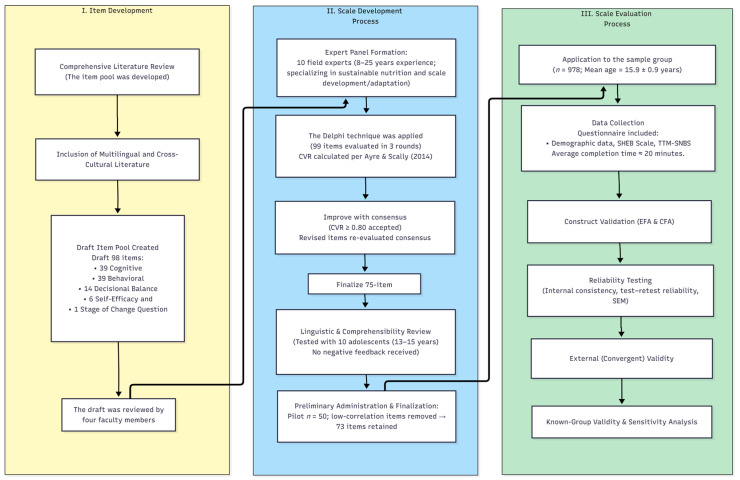
Flowchart of the Transtheoretical Model-Based Sustainable Nutrition Behavior Scale (TTM-SNBS) Development and Validation Study [41]. CVR: Content Validity Ratio; EFA: Exploratory Factor Analysis; CFA: Confirmatory Factor Analysis; SEM: Structural Equation Modeling.

In the final phase of the study, the TTM-SNBS was administered to the target sample, and psychometric analyses were conducted to establish the scale’s validity and reliability. The overall development and validation process is summarized in Figure 2.

### 2.5. Scale Evaluation

#### 2.5.1. Participants

This cross-sectional study was conducted between March and June 2023 in two public Anatolian high schools located in the Çankaya district of Ankara, Türkiye. The study population consisted of students in the 9th, 10th, and 11th grades who were aged 12 years and above, had reading comprehension and response skills, and whose mother tongue was Turkish. Individuals who were either international students or below the age of 12 were excluded from the study.

The minimum sample size required for validity and reliability testing was determined. According to the literature for internal construct reliability, at least 500 students should have scale responses [45]. For test–retest reliability, the scale was reapplied to the students one week later, and repeatability was evaluated. For an intraclass correlation coefficient (ICC) of 0.50, it was decided to reapply the TTM to at least 30 of the students with a type I error of 0.05 and 90% power [46]. For external construct validity, the required sample size was calculated to obtain a relationship of at least 0.60 (>0.50) with the Sustainable and Healthy Eating Behaviors Scale (SHEBS) with a type I error of 0.05 and 90% power was calculated as at least 510 [47].

#### 2.5.2. Data Collection

Prior to the study, students and their parents were informed about its purpose, and written consent was obtained. The questionnaire was administered to 978 adolescents who voluntarily participated, with completion taking approximately 20 min.

A data collection form consisting of general information and scales sections was used in the study. Demographic and anthropometric data were collected through the general information section. At the scales section, the validated Turkish version of the SHEBS [12] is included. SHEBS has been developed in accordance with the FAO’s sustainable diet concept, the LiveWell approach, and principles of sustainable and healthy nutrition behavior. SHEBS was used to assess sustainable dietary behaviors across eight factors. Also, the TTM-SNBS, developed to determine the gradual change in adolescents’ sustainable nutrition behaviors within the scope of this study, was applied. The TTM-SNBS was finalized with 75 items after three Delphi sessions and structured according to the core dimensions of the TTM (stages of change, cognitive and behavioral processes, decisional balance, and self-efficacy). The detailed structure of all instruments, including item counts, scale types, and scoring, is presented in Appendix A.

#### 2.5.3. Statistical Analysis

The developed 75-item draft TTM-SNBS first established content validity through expert opinion and demonstrated comprehensibility and consistent responses during a preliminary study. The comprehensive psychometric evaluation of the scale’s validity and reliability was performed in the following stages:

Exploratory Factor Analysis (EFA): Conducted to identify the factor structure of the scale. Items with standardized factor loadings ≥ 0.30 were retained, and factor retention was guided by eigenvalues > 1 and the scree plot. Kaiser-Meyer-Olkin (KMO) and Bartlett’s test of sphericity tests were used to determine the sample size and suitability for factor analysis. The proportion of variance explained was reported for the identified factors [48,49].

Confirmatory Factor Analysis (CFA): Performed to validate the final structure obtained from EFA. Model fit indices included χ^2^/*df*, Root Mean Square Error of Approximation (RMSEA), Standardized Root Mean Square Residual (SRMR), Comparative Fit Index (CFI), and Tucker–Lewis Index (TLI), with thresholds of χ^2^/*df* < 5, RMSEA ≤ 0.08, SRMR ≤ 0.08, and CFI/TLI ≥ 0.95 considered acceptable [20].

Construct Validity: The internal consistency of the scales that demonstrated construct validity was examined using Cronbach’s alpha coefficient. A coefficient value > 0.90 was considered excellent, >0.80 good, >0.70 acceptable, and >0.60 questionable in terms of reliability [50].

Test–retest Reliability: To evaluate the test–retest reliability of the scales, the ICC was calculated using a two-way mixed-effects model with absolute agreement. ICC values were interpreted as follows: below 0.50 = poor reliability, between 0.50 and 0.75 = moderate reliability, between 0.76 and 0.90 = good reliability, and above 0.90 = excellent reliability [51].

Measurement Error: The Standard Error of Measurement (SEM) was also calculated to assess the measurement error of the scales.

Construct Validity-Hypothesis Testing: To evaluate the construct validity of the TTM-SNBS, their relationships with the subdimensions of the SHEBS were examined. The following thresholds were used to interpret the correlation coefficients: <0.30 = negligible, <0.50 = low, <0.70 = moderate, <0.90 = high, and ≥0.90 = very high correlation [52].

Known-Group Validity: Assessed by comparing the scale scores of students in the highest and lowest 27% groups based on the total TTM-SNBS score using the Mann–Whitney U test.

Floor and Ceiling Effects: Considered to be present if ≥15% of participants obtained the lowest or highest possible score on the total and subscale scores of the TTM-SNBS [53].

All statistical calculations and analyses were performed using IBM SPSS Statistics version 21.0 (IBM Corp. Released 2012. Armonk, NY, USA: IBM Corp.) and the open-source R version 4.3.2 [54]. In R, the “psych” [55], “lavaan” [56], and “semPlot” [57] packages were used. The level of statistical significance was set at *p* < 0.05.

## 3. Results

### 3.1. Item Pool and Content Validity

For content validity, consensus was reached in the third round of expert review for a total of 75 items. Items that reached consensus in earlier rounds were excluded from subsequent evaluations, whereas items without consensus were revised based on expert recommendations. This process resulted in the refinement, removal, or addition of items. The content validity index (CVI) values for the entire scale ranged between 0.78 and 1.00. The final number of items per dimension, as determined by consensus, is presented in Table 1. Additionally, the items were reviewed by 10 students to assess comprehensibility, and positive feedback was received.

### 3.2. Preliminary Study Results

Following the preliminary evaluation of 50 students, the median completion time for the TTM-SNBS was 16 min (Q1 = 12, Q3 = 20). The internal consistency for all scales ranged between 0.70 and 0.94, indicating adequate reliability (Table 2). The item “I take care not to waste food and beverages” showed a negative correlation with the total score, thereby impairing summability. Similarly, in the CS-DB subscale, the item “I enjoy consuming sugary and fizzy drinks when I am thirsty” exhibited a low and negative correlation with the total score. Consequently, items demonstrating low or negative item–total correlations within the C-PCS and CS-DB subscales were removed. In total, 73 items were retained for the validity analyses.

### 3.3. Study Sample Characteristics

The study was completed with responses from 978 students, 53.8% of whom were male. The mean age of the participants was 15.9 ± 0.9 years, and the mean BMI was 21.1 ± 3.6 kg/m^2^ (Table 3).

### 3.4. Internal Construct Validity

Item–total correlation and factor structure analyses were performed separately for each subscale of the TTM-SBDS. Decisions to retain or remove items were based on item misfit in the item response theory model, low factor loadings in EFA (<0.30), and evaluations of model performance and reliability with or without the item. KMO values above 0.60 confirmed that the sample size was adequate for factor analysis. Bartlett’s test of sphericity was significant for all scales (*p* < 0.001), indicating sufficient inter-item correlations. The explained variance of the scales ranged from 37.8% to 60.2%, while internal consistency values ranged from 0.617 to 0.915. The final item numbers for all subscales, together with the EFA and CFA results, are presented in Table 4. The C-PCS was structured into three factors, the B-PCS into four factors, and the PS-DB, CS-DB, and SES each into a single factor.

**Figure 3:**
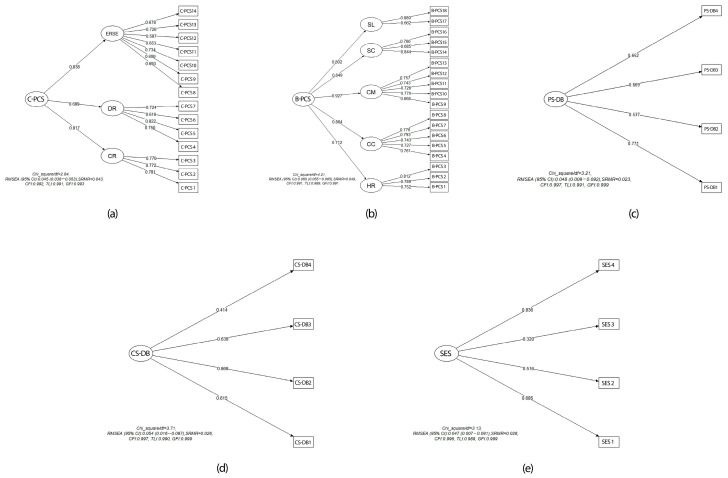
Confirmatory Factor Analysis (CFA) models of the TTM-SNBS subscales. (**a**) Second-order factor model of the Cognitive Change Process Scale (C-PCS). Environmental Reevaluation and Self-evaluation (ERSE), Dramatic Relief (DR), Consciousness Raising (CR); (**b**) Second-order factor model of the Behavioral Change Process Scale (B-PCS). Self Liberation (SL), Stimulus Control (SC), Contingency Management (CM), Counter Conditioning (CC), Helping Relationship (HR); (**c**) First-order factor model of the Pros of Decisional Balance (PS-DB); (**d**) Second-order factor model of the Cons of Decisional Balance (CS-DB); (**e**) Second-order factor model of the Self-Efficacy Scale (SES). Chi_square/*df*: Chi-square divided by degrees of freedom; RMSEA: Root Mean Square Error of Approximation; SRMR: Standardized Root Mean Square Residual; CFI: Comparative Fit Index; TLI: Tucker–Lewis Index; GFI: Goodness-of-Fit Index.

CFA was performed to validate the structure identified through EFA. Model fit indices and factor loadings obtained from CFA are presented in Figure 3a–e for each subscale. The χ^2^/*df* values were lower than the accepted limit value in the literature (<3 indicates a good fit, <5 indicates an acceptable fit). The CFI, TLI, and GFI values exceeded 0.95, indicating good model fit. The RMSEA value was below 0.08 for all subscales, further supporting model adequacy.

**Figure 4:**
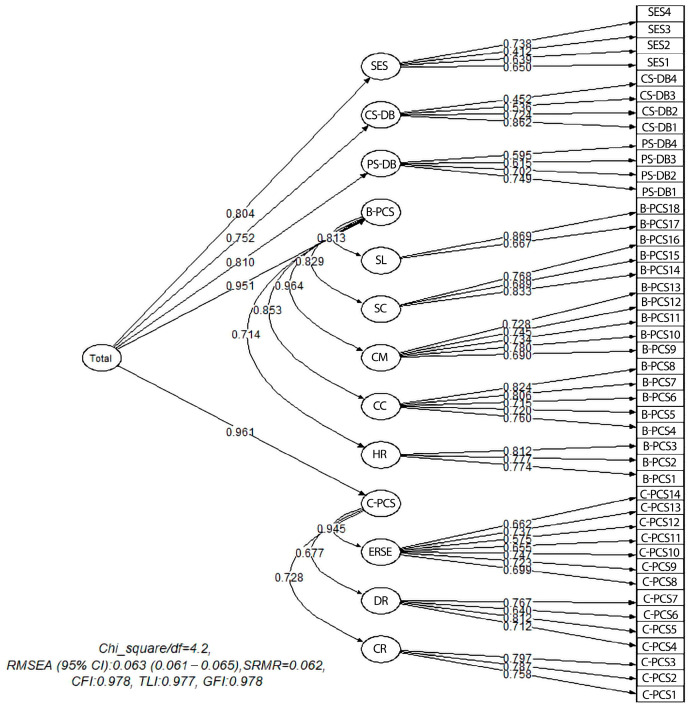
Third-order factor model in Confirmatory Factor Analysis of the total score. SES: Self-Efficacy Scale; CS-DB: Cons of Decisional Balance; PS-DB: Pros of Decisional Balance; B-PCS: Behavioral Change Process Scale; SL: Self Liberation; SC: Stimulus Control; CM: Contingency Management; CC: Counter Conditioning; HR: Helping Relationship; C-PCS: Cognitive Change Process Scale; ERSE: Environmental Reevaluation and Self-evaluation; DR: Dramatic Relief; CR: Consciousness Raising; Chi_square/*df*: Chi-square divided by degrees of freedom; RMSEA: Root Mean Square Error of Approximation; SRMR: Standardized Root Mean Square Residual; CFI: Comparative Fit Index; TLI: Tucker–Lewis Index; GFI: Goodness-of-Fit Index.

A factor analysis based on total scores across the C-CPS, B-CPS, PS-DB, CS-DB, and SES items indicated that the overall model demonstrated good fit. The corresponding model and fit index values are displayed in Figure 4.

To assess test–retest reliability, the scales were re-administered to students after one week. Inconsistent responses were excluded. The resulting ICCs indicated moderate-to-excellent repeatability (Table 5).

Convergent validity (external construct validity) was examined by analyzing the correlations between the total score of the TTM-SNBS and the subscales of the SHEBS (Table 6). All correlation coefficients were statistically significant (*p* < 0.001). The observed correlations ranged from r = 0.455 to r = 0.588, indicating low-to-moderate positive relationships between sustainable nutrition behaviors and sustainable and healthy eating behaviors. These findings provide empirical support for the convergent validity of the TTM-SNBS.

Floor and ceiling effects for all scales were below 15% (Table 7). For known-group validity, the top 27% (*n* = 262) and bottom 27% of participants were compared based on total scores. The differences between these two groups were statistically significant (*p* < 0.001). Students in the top 27% group had higher TTM-SNBS total scores compared with those in the bottom 27% group.

**Figure 5:**
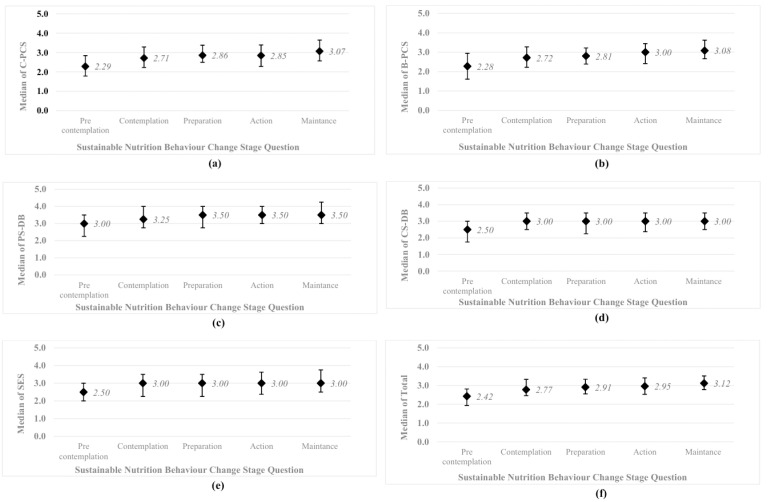
Median scores and interquartile range values of the Sustainable Nutrition Behavior Change question response categories: (**a**) Cognitive Processes of Change Scales (C-PCS); (**b**) Behavioral Processes of Change Scales (B-PCS); (**c**) Pros Scale of Decisional Balance (PS-DB); (**d**) Cons Scale of Decisional Balance (CS-DB); (**e**) Self-Efficacy Scale (SES); (**f**) TTM-SNBS Total Score.

Analysis of responses to the Sustainable Nutrition Change Stage question revealed that 27.8% of students reported not following sustainable eating patterns and not planning to change within the next six months, whereas 16.0% reported maintaining sustainable eating habits for more than six months. The distribution of total and subscale scores based on stage-of-change responses is presented in Figure 5. Statistical comparisons indicated that at least one response category differed significantly from another (all *p* < 0.001).

## 4. Discussion

This study aimed to develop and validate an original scale based on the transtheoretical model in Turkish that comprehensively assesses sustainable eating behaviors and evaluates its psychometric properties. Systematic reviews examining model-based interventions targeting nutrition behaviors indicate that the TTM is frequently used, particularly for tools related to the stages of change. However, reliable and valid measurement instruments for other constructs—processes of change, decisional balance, and self-efficacy—remain limited. The TTM-SNBS addresses this methodological gap by incorporating all four core components of the TTM.

### 4.1. Content Validation

Several studies have employed the Delphi technique to ensure content validity during scale development [37,58,59]. Similarly, in this study, the Delphi technique was applied, and item clarity was enhanced through linguistic review and student feedback following iterative rounds. The CVI values ranged from 0.78 to 1.00 across all subscales, exceeding recommended thresholds [60]. While similar scales in the literature have often relied solely on expert opinion [5,7,10,12], the TTM-SNBS demonstrated stronger content validity by combining a systematic Delphi process with feedback from the target population. Furthermore, in a preliminary assessment involving 50 students, the scale’s comprehensibility and appropriate administration time were confirmed, and items with low correlations were removed, resulting in a 73-item version for subsequent analyses.

### 4.2. Structural Validity

Both EFA and CFA confirmed the construct validity of the TTM-SNBS. KMO values (0.66–0.93) and Bartlett’s test results (*p* < 0.001) indicated suitability for factor analysis, with explained variance ranging from 37.8% to 60.2%. The analyses revealed a multidimensional factor structure reflecting the four TTM constructs (processes of change, decisional balance, self-efficacy, and stages). The meaningful and theoretically consistent factor loadings across subscales indicate that the model aligns with the conceptual framework of the TTM.

Previous studies have shown that TTM-based scales are often designed to measure a single construct [16,21]. For example, Erol et al. [17] developed a “process of change” scale for fruit and vegetable consumption among adolescents, while Kadıoğlu et al. [26] focused solely on self-efficacy. The TTM-SNBS uniquely contributes to the literature as the first Turkish scale to simultaneously validate all four core constructs of the TTM.

### 4.3. Internal Consistency

The reliability of the TTM-SNBS was evaluated in terms of internal consistency and temporal stability. Cronbach’s α coefficients ranged from 0.75 to 0.93 across subscales, with items showing low (<0.30) or negative item-total correlations removed to enhance homogeneity. These coefficients fall within the good-to-excellent range based on accepted thresholds [61] and demonstrate stronger internal consistency than those reported in previous studies [7,12].

Test–retest analysis showed significant stability across all subscales, with ICC values ranging from moderate to good and strong reliability observed for the total score [51]. Whereas most previous studies reported reliability only for the stage construct in TTM-based scales [27], this study also provided reliability evidence for the decisional balance, processes, and self-efficacy dimensions.

### 4.4. External Validity, Known Groups, and Ceiling/Floor Effects

External validity was examined using the SHEBS, which measures sustainable and healthy eating behaviors. Significant correlations in the expected direction were found between the TTM-SNBS and the SHEBS subscales (r = 0.26–0.58), supporting conceptual overlap while also highlighting the unique structure of the TTM-SNBS. While the literature generally reports only moderate correlations for stage-related constructs in TTM-based scales [17,26,27], the present findings provide evidence for the external validity of all four constructs.

Known-group validity analyses revealed significant differences between the lowest and highest 27% groups (*p* < 0.001). In addition, the ceiling and floor effects remained below 15% for all subscales, indicating that the scale retained strong discriminative ability and sensitivity across score distributions.

### 4.5. Contributions, Limitations, and Future Studies

This scale not only serves a descriptive purpose but also provides a dynamic tool for tracking behavioral change and planning interventions. In this respect, it fills a critical gap, particularly in education-based behavior change programs.

A major strength of this study is its adherence to COSMIN guidelines. The use of multiple validity assessments combined with a large sample size enhances the reliability and generalizability of the findings.

However, several limitations should be acknowledged. Since the sample was limited to high school students, there is a risk of selection bias, which restricts generalization to other age groups or socioeconomic backgrounds. Additionally, self-reported measures may introduce social desirability bias, and caution should be exercised when generalizing these findings.

Another potential limitation is that the concept of sustainable nutrition may vary across sociocultural contexts. As the TTM-SNBS was developed and validated among Turkish adolescents, future studies should examine its cross-cultural applicability and measurement invariance in different adolescent populations. Further studies are encouraged to validate the scale in other populations and conduct additional reliability and validity analyses across diverse cultural settings.

In practical terms, the TTM-SNBS offers a valuable instrument for educators, researchers, and policymakers to design, implement, and evaluate theory-based interventions promoting sustainable dietary behaviors among adolescents. This study demonstrates that the proposed framework can effectively guide nutrition education and public health initiatives aimed at fostering sustainability.

## 5. Conclusions

This study developed and validated the TTM-SNBS for adolescents. The scale demonstrated strong psychometric properties, including robust content, construct, and external validity, along with good reliability across subscales. Unlike previous instruments focusing on a single construct, the TTM-SNBS provides a comprehensive framework for assessing sustainable nutrition behaviors by integrating all four core components of the TTM—stages of change, processes of change, decisional balance, and self-efficacy. By offering a reliable and valid instrument, this study fills a methodological gap and contributes to advancing theory-based assessment in nutrition behavior research. Future studies should investigate the applicability of the TTM-SNBS across diverse populations and cultural contexts to further strengthen its generalizability and practical utility in intervention planning.

## Figures and Tables

**Table 1:** Number of Items with Expert Consensus and Content Validity Indices for Each Subscale of the TTM-SNBS.

Subscale of the TTM-SNBS	Number of Items with Expert Consensus	Content Validity Index (CVI)
Stages of Change Questionnaire (SCQ)	1	1.00
Cognitive Processes of Change Scale (C-PCS)	31	0.78–1.00
Behavioral Processes of Change Scale (B-PCS)	26	0.78–1.00
Pros Subscale of Decisional Balance (PS-DB)	4	0.78–1.00
Cons Subscale of Decisional Balance (CS-DB)	7	0.78–1.00
Self-Efficacy Scale (SES)	6	0.78–1.00
Total	75 items	

**Table 2:** Internal Consistency Values Obtained from the Preliminary Study (*n* = 50).

Subscale of the TTM-SNBS	Number of Items	Cronbach’s Alpha	Item–Total Correlation (*p*-Value)
SCQ	1	-	-
C-PCS	31	0.91	0.003
	30 *	0.92	0.222
B-PCS	26	0.94	0.124
PS-DB	4	0.72	0.119
CS-DB	7	0.72	0.328
	6 ^&^	0.79	0.155
SES	6	0.70	0.456

* The item “I take care not to waste food and beverages’ was removed. ^&^ The item “Enjoying sugary and fizzy drinks when thirsty” was removed.

**Table 3:** Descriptive Characteristics of the Participants (*n* = 978).

Variable	*n* (%) or Median (Q1–Q3)	Variable	*n* (%) or Median (Q1–Q3)
Sex	*n* = 960	Living arrangement	*n* = 953
Male	516 (53.8)	With family	920 (96.5)
Female	444 (46.2)	Person in family (Median(Q1–Q3))	4 (4–5)
		With a family member	28 (3.0)
Age (years)	*n* = 952	In a dormitory	5 (0.5)
Mean ± SD Median (Q1–Q3)	15.9 ± 0.9 15.9 (15.2–16.6)	Median no. of employed individuals in a household	2 (1–2)
BMI (kg/m^2^)	(*n* = 942)	Currently employed parent(s)	
Mean ± SD Median (Q1–Q3)	21.1 ± 3.6 20.5 (18.7–22.8)	Mother Father	418 (42.7) 840 (85.9)
Grade level	*n* = 965		
9th grade	344 (35.6)		
10th grade	338 (35.0)		
11th grade	283 (29.4)		
Education			
Mother’s education	(*n* = 954)	Father’s education	(*n* = 949)
Primary school	102 (10.7)	Primary school	73 (7.7)
Middle school	129 (13.5)	Middle school	114 (12.0)
High school	420 (44.0)	High school	379 (40.0)
University	255 (26.8)	University	319 (33.6)
Postgraduate	48 (5.0)	Postgraduate	64 (6.7)

*n* (%): frequency (percentage); SD: standard deviation; Q1–Q3: Quartile 1–Quartile 3; BMI: Body Mass Index.

**Table 4:** Results of Construct Validity Analysis.

Scale	N. of Baseline Items	N. of Remaining Items	Cronbach Alpha	KMO	Cumulative Variance	χ^2^/*df*	RMSEA 95% CI Lower-Upper	SRMR	CFI
C-PCS	30	14	0.89	0.91	52.4	2.8	0.045 (0.038–0.053)	0.043	0.992
B-PCS	26	18	0.93	0.93	60.2	4.2	0.060 (0.055–0.065)	0.049	0.991
PS-DB	4	4	0.75	0.75	43.8	3.2	0.048 (0.009–0.092)	0.023	0.997
CS-DB	6	4	0.72	0.69	42.4	3.7	0.054 (0.016–0.097)	0.026	0.997
SES	6	4	0.67	0.66	37.8	3.1	0.047 (0.007–0.091)	0.026	0.996

N.: number; KMO: Kaiser-Meyer-Olkin measure of sampling adequacy; χ^2^/*df*: Chi-Square/degrees of freedom; RMSEA: Root Mean Square Error of Approximation; SRMR: Standardized Root Mean Square Residual; CFI: Comparative Fit Index; C-PCS: Cognitive Processes of Change Scale; B-PCS: Behavioral Processes of Change Scale; PS-DB: Pros Subscale of Decisional Balance; CS-DB: Cons Subscale of Decisional Balance; SES: Self-Efficacy Scale. Cronbach’s alpha value > 0.90 was considered excellent, >0.80 good, >0.70 acceptable, and >0.60 questionable [50].

**Table 5:** Test–retest reliability.

TTM-SNBS	*n*	First Evaluation Median (Q1–Q3)	Second Evaluation Median (Q1–Q3)	ICC (95% CI Lower-Upper)	Standard Error of Measurements $\left(SEM=SD\times \sqrt{\left(1-ICC\right)}\right)$
C-PCS	58	2.68 (2.27–3.22)	2.82 (2.50–3.30)	0.780 (0.640–0.867)	0.34
B-PCS	52	2.67 (2.28–3.17)	2.97 (2.46–3.39)	0.783 (0.631–0.874)	0.32
PS-DB	55	3.25 (2.75–3.75)	3.00 (2.75–3.75)	0.699 (0.536–0.812)	0.46
CS-DB	48	3.00 (2.00–3.44)	2.75 (2.50–3.25)	0.584 (0.360–0.744)	0.50
SES	57	2.50 (2.25–3.25)	3.00 (2.50–3.25)	0.707 (0.550–0.816)	0.43
Total	60	2.76 (2.39–3.06)	2.92 (2.51–3.29)	0.813 (0.678–0.891)	0.26

C-PCS: The Cognitive Processes of Change Scales; B-PCS: The Behavioral Processes of Change Scales; PS-DB: The Pros Scale of Decisional Balance; CS-DB: The Cons Scale of Decisional Balance; SES: Self-Efficacy Scale n: sample size; Q1–Q3: Quartile 1–Quartile 3; ICC: Intraclass Correlation Coefficient, two-way mixed model, absolute agreement; CI: Confidence Interval; SEM: Standard Error of Measurement; SD: Standard Deviation of Measurement. An ICC value below 0.50 was considered to indicate poor reliability, a value between 0.50 and 0.75 as moderate reliability, a value between 0.76 and 0.90 as good reliability, and a value above 0.90 as excellent reliability [51].

**Table 6:** The correlation coefficient between TTM-SNBS and the SHEBS (external validity).

SHEBS	C-PCS	B-PCS	PS-DB	CS-DB	SES	Total
Quality Labels (Local and Organic)	0.479	0.579	0.436	0.338	0.424	0.588
Seasonal Foods and Avoiding Food Waste	0.408	0.488	0.424	0.303	0.424	0.513
Animal Health	0.455	0.485	0.358	0.283	0.330	0.507
Reducing Meat Consumption	0.436	0.432	0.322	0.298	0.400	0.481
Healthy and Balanced Nutrition	0.449	0.527	0.377	0.292	0.378	0.536
Local Food	0.408	0.449	0.262	0.285	0.268	0.455
Low Fat	0.396	0.456	0.390	0.267	0.367	0.479
Quality Labels (Local and Organic)	0.479	0.579	0.436	0.338	0.424	0.588

*n* = 790, Spearman rho correlation coefficient, all *p*-values < 0.001. C-PCS: The Cognitive Processes of Change Scales; B-PCS: The Behavioral Processes of Change Scales; PS-DB: The Pros Scale of Decisional Balance; CS-DB: The Cons Scale of Decisional Balance; SES: Self-Efficacy Scale. r < 0.30: negligible, <0.50: low, <0.70: moderate, <0.90: high, ≥0.90: very high correlation [52].

**Table 7:** Floor and ceiling effect.

TTM-SNBS	Floor Effect (%)	Ceiling Effect (%)
C-PCS	0.72	0.41
B-PCS	2.07	0.72
PS-DB	2.07	3.83
CS-DB	5.59	2.48
SES	3.93	2.90
Total	0.62	0.21

C-PCS: The Cognitive Processes of Change Scales; B-PCS: The Behavioral Processes of Change Scales; PS-DB: The Pros Scale of Decisional Balance; CS-DB: The Cons Scale of Decisional Balance; SES: Self-Efficacy Scale. The ratio of those with the highest and lowest scores above 15% was interpreted as floor and ceiling effects [53].

## Data Availability

The data presented in this study are available on request from the corresponding author. The data are not publicly available due to ethical restrictions.

## Abbreviations

The following abbreviations are used in this manuscript

SDGs	Sustainable Development Goals
WHO	World Health Organization
FAO	Food and Agriculture Organization
TTM	Transtheoretical Model
TTM-SNBS	Transtheoretical Model-based Sustainable Nutrition Behavior Scale
CVR	Content Validity Ratio
REDCap	Research Electronic Data Capture
COSMIN	COnsensus-based Standards for the selection of health Measurement INstruments
SHEBS	Sustainable and Healthy Eating Behaviors Scale
EFA	Exploratory Factor Analysis
CFA	Confirmatory Factor Analysis
CFI	Comparative Fit Index
SEM	Structural Equation Modeling
ICC	Intraclass Correlation Coefficient
SCQ	Stages of Change Questionnaire
C-PCS	Cognitive Processes of Change Scale
ERSE	Environmental Reevaluation and Self-evaluation
DR	Dramatic Relief
CR	Consciousness Raising
B-PCS	Behavioral Processes of Change Scale
SL	Self Liberation
SC	Stimulus Control
CM	Contingency Management
CC	Counter Conditioning
HR	Helping Relationship
PS-DB	Pros Subscale of Decisional Balance
CS-DB	Cons Subscale of Decisional Balance
SES	Self-Efficacy Scale
Chi_square/*df*	Chi-square divided by degrees of freedom
RMSEA	Root Mean Square Error of Approximation
SRMR	Standardized Root Mean Square Residual
TLI	Tucker–Lewis Index
GFI	Goodness-of-Fit Index

## Appendix A

### Data Collection Form

This appendix presents a detailed structure of the tools used in the study, including item counts, scale type, scoring, and subdimensions.

**General Information Section**				
	**Description**	**Number of Items/Questions**	**Scale Type**	**Subdimensions/Notes**
General Information	Collects demographic and anthropometric data.	10 questions	Nominal/ Continuous	Gender, age, date of birth, height, weight, household, parental info
Scales Section				
SHEBS (Köksal et al., 2023) [12]	Sustainable and Healthy Eating Behaviors Scale	34 items (in 8 factors)	7-point Likert	8 factors: Healthy nutrition, Quality marks, Meat reduction, Local food, Low fat, Avoiding waste, Animal health, Seasonal food
TTM-SNBS	Transtheoretical Model-Based Sustainable Nutrition Behavior Scale	1 question & 75 items (After three Delphi sessions)	Categorical & 5-point Likert	Structured according to TTM dimensions: stage of change question, change process (cognitive & behavioral), decision balance (pros & cons), and self-efficacy.
Subdimensions of the TTM-SNBS				
SCQ	Stages of Change Questionnaire	1 question	Categorical (5 stages)	Stages: Precontemplation, Contemplation, Preparation, Action, Maintenance
C-PCS	Cognitive Processes of Change Scale	31 items	5-point Likert	Higher scores indicate greater awareness.
B-PCS	Behavioral Processes of Change Scale	27 items	5-point Likert	Higher scores indicate stronger behavioral engagement.
PS-DB	Pros Subscale of Decisional Balance	4 items	5-point Likert	Higher scores reflect higher perceived benefits
CS-DB	Cons Subscale of Decisional Balance	7 items	5-point Likert	Higher scores reflect higher perceived barriers
SES	Self-Efficacy Scale	6 items	5-point Likert	Higher scores indicate stronger self-efficacy
